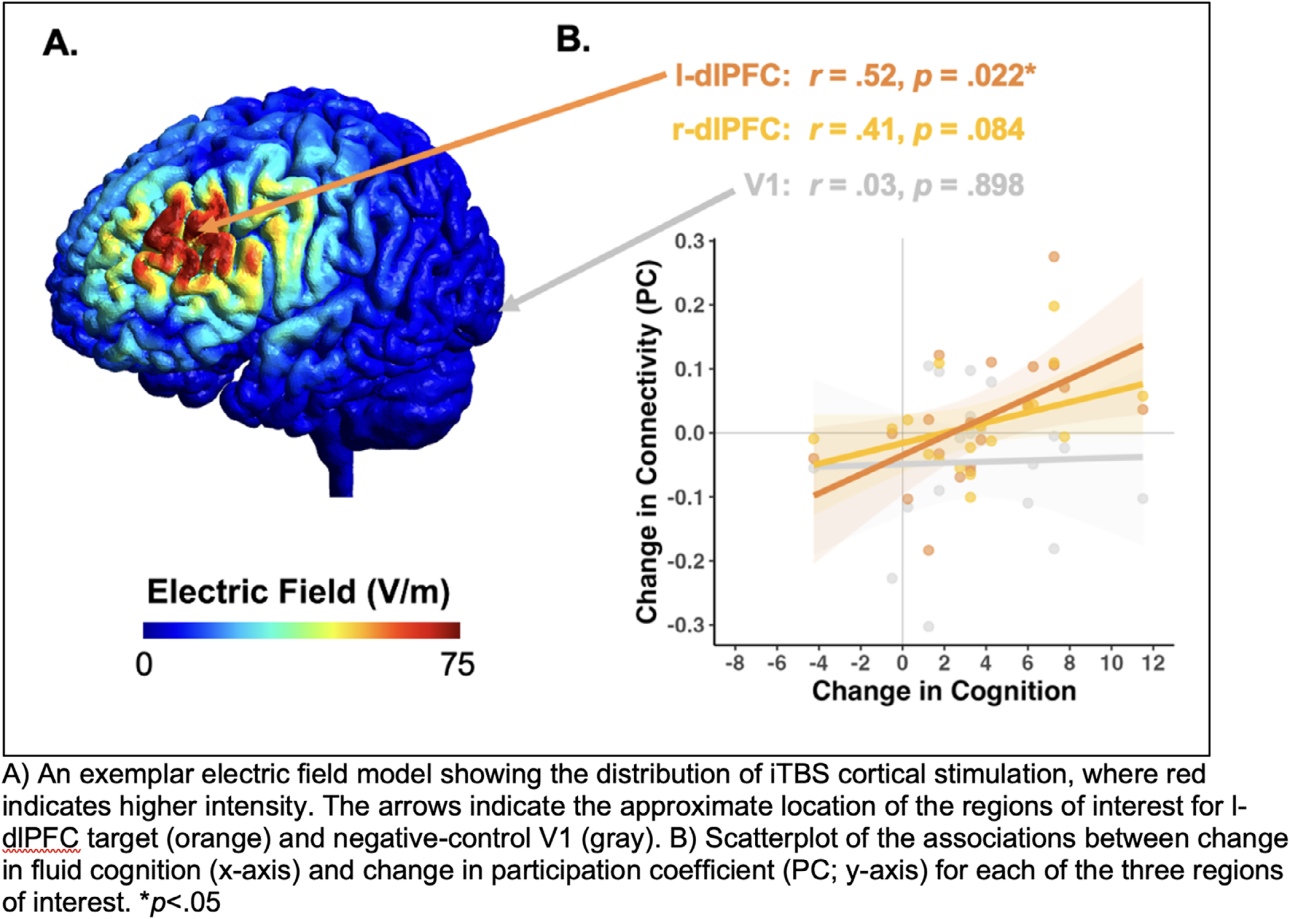# Increased Functional Connectivity Relates to Cognitive Improvement Following Non‐Invasive Brain Stimulation in MCI

**DOI:** 10.1002/alz.093289

**Published:** 2025-01-09

**Authors:** Stephanie Aghamoosa, Sara Nolin, Andrew A Chen, Kevin A Caulfield, James Lopez, Katrina Rbeiz, Holly H Fleischmann, Olivia Horn, Katrina Madden, Michael U Antonucci, Gonzalo Revuelta, Lisa M McTeague, Andreana Benitez

**Affiliations:** ^1^ Medical University of South Carolina, Charleston, SC USA

## Abstract

**Background:**

Repetitive transcranial magnetic stimulation enhances cognition in people with mild cognitive impairment (MCI). Whereas conventional treatment requires daily sessions for 4‐6 weeks, accelerated intermittent theta burst stimulation (iTBS) shortens the treatment course to just 3 days, substantially improving feasibility of use in people with MCI. We conducted a Phase I safety and feasibility trial of iTBS in MCI, finding preliminary evidence of cognitive improvement. Here, we explore the neural mechanism of this effect by evaluating iTBS‐related changes in functional connectivity in relation to the observed cognitive change.

**Method:**

Twenty‐two patients with amnestic MCI received iTBS to left dorsolateral prefrontal cortex (l‐dlPFC) over 3 treatment days (with 8 stimulation sessions of 600 pulses per day) within 1 week. Nineteen had complete MRI and cognitive testing data. The primary cognitive outcome was the fluid cognition composite score from the NIH Toolbox Cognition Battery. We computed functional connectivity from resting‐state fMRI and calculated participation coefficient for three regions of interest. Lower participation coefficient values indicate that a region is more selectively connected to its own network and higher values indicate that it is more widely connected across networks (i.e., a “hub”). We calculated change in participation coefficient from pre‐ to post‐treatment for two regions belonging to the frontoparietal network (the l‐dlPFC iTBS target and the contralateral right dlPFC) and a negative control (primary visual; V1).

**Result:**

There was a significant, large effect‐size (d = 0.98) improvement in fluid cognition from pre‐ to post‐iTBS treatment. Improvements in cognition were significantly associated with increased participation coefficient of l‐dlPFC (*r* = .52, *p* = .022), with a marginal effect in r‐dlPFC (*r* = .41, *p* = .084) and a near zero effect in the negative control V1 (*r* = .03, *p* = .898).

**Conclusion:**

This preliminary investigation suggests that iTBS‐related cognitive improvement in MCI may be attributable to increased connectivity of the stimulated frontoparietal network. Specifically, a larger increase in “hubness” (i.e., connectivity across multiple networks) of the l‐dlPFC target region is associated with greater cognitive gains. However, as these analyses are based on a limited number of regions and small sample, future studies are needed to further evaluate the neural mechanisms of iTBS in MCI.